# Compassion Fatigue Experiences of Organ Transplant Nurses: A Qualitative Method

**DOI:** 10.1002/nop2.70561

**Published:** 2026-04-29

**Authors:** Mediha Ekici, Nilgün Aksoy

**Affiliations:** ^1^ Organ Transplantation Unit, Akdeniz University Hospital Antalya Turkey; ^2^ Faculty of Nursing, Akdeniz University Antalya Turkey

**Keywords:** compassion, compassion fatigue, nursing, qualitative research, transplant nursing

## Abstract

**Aim:**

This study aimed to explore and understand the compassion fatigue experiences of organ transplant nurses.

**Design:**

This study employed the phenomenological design, which is a qualitative research method.

**Method:**

The data of the study were collected from 13 nurses working at the Organ Transplant Clinics of a University Hospital in southern Turkey. A “Personal Information Form” and a “Semi‐Structured Interview” form were used to collect data in face‐to‐face in‐depth interviews held between December 2022 and June 2023. The content analysis method was used to analyze the data.

**Results:**

In this study, 248 codes, 11 categories, and three themes were obtained. The themes were “Causes of Compassion Fatigue”, “Symptoms of Compassion Fatigue”, and “Management of Compassion Fatigue”.

**Conclusion:**

It was determined that all participants experienced compassion fatigue. The factors that contributed to the development of compassion fatigue were the high amount of care burden in the care of organ transplant patients, the insufficient number of nurses, having children, and rejection cases, whereas preventive factors included raising awareness regarding the development of compassion fatigue and the creation of personal care strategies.

**Reporting Method:**

This research has adhered to relevant EQUATOR guidelines, the consolidated criteria for reporting qualitative studies (COREQ 32‐item checklist).

**Patient or Public Contribution:**

Thirteen participants with at least a bachelor's degree in transplant nursing completed the research.

## 1 Introduction

Organ transplantation is an innovative field that has pioneered research as a result of the development of surgical techniques in the mid‐20th century, advancements in the understanding of the immunological processes causing organ rejection, the initiation of drug therapies used to prevent organ rejection, and the establishment of organ preservation techniques (Yussim and Mor 2023). It is the best treatment option for patients to lead normal lives and have increased levels of expected quality of life (Horka 2022).

In the organ transplantation process, a multidisciplinary team approach is needed to achieve optimal outcomes for both the donor and the recipient (Horka 2022; Ionescu et al. 2022). Recent developments in post‐transplant care have led to an increase in the life expectancy of organ transplant recipients (Salam et al. 2019). However, despite improvements in general graft functioning and survival rates after solid organ transplantation, complications may lead to severe morbidity and mortality (Sen et al. 2019). Organ transplant recipients have complex healthcare requirements, and they need to be successful in self‐management and receive consistent care and patient education (Mangold 2020). Nursing care has critical importance that involves the physical, psychological, and social aspects of patients (Guichard et al. 2021).

Compassion is the foundation of palliative care and is considered the main building block of high‐quality care (Skorpen Tarberg et al. 2020). It is a concept that refers to the intense emotion that arises when a person faces the pain of another person and desires to alleviate that pain (Goldberg 2020). The literature indicates that compassion fatigue develops over time as a result of continuous and repetitive contact with individuals exposed to trauma (Figley 1995). Compassion fatigue is characterized by the development of emotional, physical, and mental burnout as a result of working with traumatized persons (Alharbi et al. 2019). Nursing is a high‐risk and stressful profession, and this situation leads nurses to have increased susceptibility to the symptoms of compassion fatigue in comparison to other healthcare personnel (Yee 2023). In the literature, it was reported that nurses who worked with chronic patients for prolonged durations were at risk of compassion fatigue and burnout (Karabuga Yakar et al. 2023; Pehlivan Saribudak and Aydın 2024). Determining obstacles to the provision of compassionate care will help nurses plan better and more appropriate interventions (Babaei and Taleghani 2019). Nurses experiencing high levels of compassion fatigue were found to have higher rates of making mistakes, providing low‐quality care, and intentions to leave their jobs (Jo et al. 2023).

Very little is known about how compassion fatigue affects a nurse as a person, and indicators of how this affects the profession as a whole are also lacking (Gustafsson and Hemberg 2022). There is a gap in the literature regarding recent studies on the compassion fatigue experiences of organ transplant nurses. Transplant nurses, provide care for patients with life‐threatening conditions requiring intensive and prolonged monitoring, are involved in emotionally challenging processes such as organ rejection and uncertain prognoses, and establish close, long‐term relationships with patients and their families during highly critical periods. While compassion fatigue may be present across nursing fields, these unique stressors may intensify emotional burden in transplant settings. In this context, this study aims to reveal the compassion fatigue experiences of organ transplant nurses in in‐depth interviews. It is believed that the results of this study will contribute to the literature in this field by offering evidence based on the views and actual lived experiences of organ transplant nurses.

## 2 Methods

### 2.1 Design

In this study, to determine the compassion fatigue experiences of organ transplant nurses, a phenomenological design and the content analysis approach were used as qualitative research methods. Qualitative research methods allow researchers to investigate the experiences of individuals in detail (Patton 2020). In phenomenological studies, it is aimed to comprehend the essence of a phenomenon by collecting information from groups that have experienced the phenomenon as a whole (Çarpar 2020). The reporting of the results of this study is based on the COREQ Checklist.

### 2.2 Participants and Settings

The population of the study consisted of nurses working at Organ Transplant clinics at a University Hospital in southern Turkey. Nurses working in the transplant clinics where the study was conducted provide care to both adult and paediatric patients. Criterion sampling, which is a purposive sampling method, was used to select participants. The criteria were created by the researchers. The target population included nurses who worked at Organ Transplant clinics and provided care to organ transplant patients. The inclusion criteria were as follows: (1) agreeing to participate in the study and providing written informed consent and (2) having worked at the organ transplant clinic for at least 6 months. To ensure data diversity, the participants were selected through purposive sampling by considering factors such as age, experience in the profession, and experience as an organ transplant nurse.

One of the approaches recommended regarding sample size in qualitative studies is the “data saturation point” at which no new information is obtained, or the collected information starts to repeat (Polit and Beck 2017). In this study, data saturation was reached in the 12th interview. However, a 13th interview was also held to further demonstrate data saturation. The study was completed with the participation of 13 nurses.

### 2.3 Data Collection

The data collection process took place between December 2022 and June 2023 using a semi‐structured interview form (Table 1) in face‐to‐face in‐depth interviews.

**TABLE 1:** Interview Guide.

Main and additional questions
Can you define transplant nursing? How would you rate the unit you work in as a transplant nurse? How does it make you feel to care for transplant patients? Which patient (kidney transplant, liver transplant, paediatric patient, donor, cadaver transplant recipient, etc.) do you find most difficult to care for? Why? How do you feel when transplant patients you care for experience a negative situation? What do compassion and compassion fatigue mean to you when caring for transplant patients? What are your experiences with compassion fatigue as an organ transplant nurse? If you were to compare your experience of compassion fatigue as an organ transplant nurse to something, what would you compare it to? Why?

The first researcher contacts each participant individually and scheduled dates and hours for interviews. The interviews were held in a meeting room used for education sessions and meetings at the Organ Transplantation Center. At the beginning of the interview, the participant was informed about the objective of the study and given explanations about the conditions of the interview such as the principle of volunteerism, the privacy of their identifying information, the audio recording process, the verbatim transcription of the audio of the interview, and the codename to be assigned to them to protect their identity during the reporting process. A semi‐structured interview form, consisting of basic open‐ended questions that allowed the participants to express their experiences in detail, was used to conduct the interviews. Each participant was directed an initiation question to start the interview. The participant was asked to define organ transplant nursing. The interview continued with in‐depth questions serving the objective of the study. The process was completed by asking an exit question and a question about metaphors the participant would use to describe compassion fatigue. The audio recordings were labelled, numbered, and securely stored on an external hard drive for data management and analysis. The shortest interview lasted 19 min, and the longest interview lasted 30 min, with an average interview duration of 26.1 ± 4.05 min.

### 2.4 Data Analysis

Because studies on the current theory or phenomenon were limited, to describe the phenomenon in question in depth, an inductive process and the qualitative content analysis method were used in this study. In qualitative content analysis, researchers aim to make the voices of their participants heard (Lindgren et al. 2020). The main steps of content analysis are coding the data, identifying themes, organizing the codes and the themes, reviewing the data in light of the codes and themes, and presenting and interpreting findings (Sığrı 2021). The content analysis in this study was performed by following the steps recommended by Creswell (2017): preparation of data for analysis, reading of all data, data coding, theme identification, making theme connections, and interpreting themes. After each interview, ME (♀) transcribed the audio recordings of the in‐depth interview in the recording device and turned them into a written document. The non‐verbal gestures and bodily expressions of the participants were also indicated in the transcripts. First, the transcripts were read independently by ME and NA to become familiar with the text and have a general understanding of the content. The researchers carried out the coding process by identifying the repeated aspects of the data and relevant themes, and they held regular meetings to discuss their findings. The codes and categories were directly obtained from the raw data. During the organization process, all meaningful statements were identified, clearly coded, and summarized by adding comments and headings to the text. At the end of the coding process, 248 codes were obtained in total. The researchers initially compared the codes in two independently reviewed transcripts and discussed their findings. Afterward, they coded the rest of the transcripts independently and created tentative themes. Meetings were held for the researchers to compare their tentative themes. The identified themes were highly consistent between the researchers. Using an inductive approach, the connections between the concepts identified through coding were revealed, the similarities and differences between expressions were found, and categories and themes were determined. As a result of the content analysis of the interviews and observations, 11 categories and 3 themes emerged. To allow the reader to make direct comparisons between the data and findings, the data of the participants were reported without adding any comments or making any changes. Finally, the emerging themes and patterns were presented.

### 2.5 Rigour and Quality Criteria

In qualitative studies, validity and reliability are associated with the credibility, repeatability, trustworthiness, transferability, and confirmability of the data (Arastaman et al. 2018). To achieve validity and reliability in such studies, the concepts of credibility (internal validity), transferability (external validity), confirmability (internal reliability), and consistency (external reliability) are utilized (Arslan 2022). In this study, to increase internal validity, a conceptual framework about the topic was created based on the review of the relevant literature while creating the Semi‐Structured Interview Form. The data were collected using the interview method. The data of the interview were summarized for each participant immediately after each interview, and whether the summary of the interview reflected their expressions was confirmed. Non‐verbal cues expressing the emotions of the participants, such as their tone of voice, gestures, and facial expressions, were recorded and used during the analyses to help researchers interpret their experiences, perceptions, views, and ideas.

To ensure external validity, the participants were selected by criterion sampling, which is a purposive sampling method, and the interviews were held based on the principle of volunteerism. Information about the sample, as well as the data collection and analysis stages, was presented in detail. Well‐written explanations were made about the settings in which the study was carried out, the characteristics of the participants, and the themes and categories obtained as findings of the study. Additionally, the detailed research and data analysis process, the depth of the explanations, and their breadth supported the confirmability and transferability of the results of the study. To ensure the internal reliability of the study, the data were reported verbatim and without adding any comments. The data collected in the interviews were coded separately by the researchers. After this, themes were created independently by the researchers and finalized by reaching a consensus. After the identification of the codes and themes, they were evaluated by two experts who were not involved in the study, and the interrater agreement ratio (Kappa value) was calculated. Kappa values between 0.81 and 1.00 are considered to indicate excellent agreement (Landis and Koch 1977). The Kappa value was calculated as 1.00 in this study (*K* = 1.00; *p* < 0.001). This value indicated an excellent rate of agreement between the coders. To ensure external reliability, the data were presented in a confirmable and clear manner. To increase external reliability, the data collection instruments, raw data, and coding processes were evaluated by an expert outside the research team.

## 3 Results

The demographic characteristics of the participants are given in Table 2. 13 participants were included in the study, and the majority of the participants (*n* = 12) were women. The mean age of the participants was 32.15 ± 5.32, and all participants had at least undergraduate university degrees. Their mean duration of working as nurses was 9 ± 5.19 years, while their mean duration of working at the Organ Transplant Clinic was 5.31 ± 4.37 years.

**TABLE 2:** Descriptive characteristics of nurses.

Participant	Age	Gender	Marital status	Number of children	Education	Income status	Working time as a nurse (Year)	Working time in organ transplant clinic (Year)	Weekly working hours
P1	35	Female	Married	2	Undergraduate	Medium	11	11	40
P2	31	Female	Single	—	Undergraduate	Low	7	5	40–48
P3	42	Female	Married	1	Undergraduate	Medium	18	11	40–48
P4	31	Female	Single	—	Undergraduate	Medium	7	5	40–48
P5	36	Female	Married	2	Undergraduate	Medium	13	4	40–48
P6	32	Female	Married	2	Undergraduate	Medium	8	5	40–48
P7	26	Female	Single	—	Undergraduate	Medium	3	1	40–48
P8	41	Female	Married	2	Master	Medium	19	15	40
P9	31	Female	Single	—	Undergraduate	Medium	8	3	40–48
P10	29	Male	Single	—	Undergraduate	Medium	6	1	40–48
P11	30	Female	Single	—	Undergraduate	Medium	7	3	40–48
P12	31	Female	Married	2	Undergraduate	Medium	9	4	40–48
P13	23	Female	Single	—	Undergraduate	Medium	1	1	40–48

Based on the data collected in the study, 248 codes, 11 categories, and three themes were obtained. The four categories under the theme “Causes of Compassion Fatigue” were “heavy burden brought about by the responsibility of the transplanted organ, shift from empathy to sympathy, getting used to stress, and heavy burden of working conditions”. The four categories under the theme “Symptoms of Compassion Fatigue” were “feeling of being unable to do anything, thoughts of inadequacy, putting oneself in the shoes of the helpless, and price of trying to do more”. The three categories under the theme “Management of Compassion Fatigue” were “improving team spirit, designing individualized care, and embracing organ transplant nursing” (Table 3).

**TABLE 3:** Themes and categories of the study.

Themes	Categories
Causes of compassion fatigue	Heavy burden brought about by the responsibility of the transplanted organ
	Shift from empathy to sympathy
	Getting used to stress
	Heavy burden of working conditions
Symptoms of compassion fatigue	Feeling of being unable to do anything
	Thoughts of inadequacy
	Putting oneself in the shoes of the helpless
	Price of trying to do more
Management of compassion fatigue	Improving team spirit
	Designing individualized care
	Embracing organ transplant nursing

Participants' metaphors for compassion fatigue experience are given in Table 4.

**TABLE 4:** Metaphors for nurses' experience of compassion fatigue.

Participants	Metaphors
P1	Swimming in a choppy sea
P2	A puppet
P3	A flower that starts to fade
P4	A balloon
P5	A mother's fatigue
P6	A baby that cries all the time
P7	Falling into a pit
P8	Being a mother
P9	A thirsty flower
P10	Race horses
P11	Walking on the road
P12	Ashes in a jar
P13	A polar bear

### 3.1 Theme: Causes of Compassion Fatigue

The causes of compassion fatigue among the participants were examined under four categories within the scope of this main theme: heavy burden brought about by the responsibility of the transplanted organ, shift from empathy to sympathy, getting used to stress, and heavy burden of working conditions.

#### 3.1.1 Heavy Burden Brought About by the Responsibility of the Transplanted Organ

The participants acknowledged that an organ for transplantation is not found easily and stated that they made extra efforts to preserve these organs.

It is a very important surgery; after all, [someone is] giving an organ, and finding the organ is important for both donors and recipients. This is why you work more carefully and selflessly … (P3)

When that organ is transplanted into their body, we feel we have responsibility about that organ, too. Urinary output may fluctuate in some patients. One inevitably feels responsible and follows [patient outcomes] more meticulously. We even call [the hospital] from our homes when needed, for example. What is the state of the patient? Did they come back from surgery? How is their urinary output?… (P12)

#### 3.1.2 Shift From Empathy to Sympathy

In general, the participants stated that they showed more compassion toward patients in the organ transplantation process as these patients still required long‐term care after a period of chronic illness.

They are in the hospital environment and have struggled for a long time. Some of them get transplants; they have many, unending problems. We are human, we have feelings of compassion in us, and we cannot get rid of these feelings. …but these feelings remain in us and affect our psychology significantly … (P3)

…I inevitably feel emotionally connected with each patient. Of course, there are patients we sympathize with rather than empathize with… Our feelings of compassion grow even further for patients who get transplants after a long time, those who get transplants at young ages, or those whose care is challenging and who have no relatives. I feel more emotionally connected to them … (P12)

I feel very sad. In fact, we should feel empathy, not sympathy. …but mine [my emotions] resemble sympathy more. I get sad. (P9)

#### 3.1.3 Getting Used to Stress

The challenging process experienced by organ transplant patients had negative reflections on the participants such as stress, weight gain, negative effects on their social life, worries, mental fatigue, and anxiety.

When I started the organ transplant clinic, I weighed 50 kg. Then—I think because of stress—I gained weight and reached 65 kg. I feel a bit more exhausted at this department. (P2)

Of course, this affects our social lives. Stress develops in connection to that exhaustion. … as a negative outcome, I experience anxiety. Anxiety and stress, in turn, affect my social life. [There is] tension, stress… (P12)

I was not this way before I started the profession. I have become a more anxious person. (P3)

…you feel worn down; you know… You may sometimes feel anxiety even when you come back home from the hospital in some cases … (P6)

#### 3.1.4 Heavy Burden of Working Conditions

According to the participants, the insufficient number of nurses and their heavy workload triggered compassion fatigue.

The number of nurses is insufficient in general, which is why we are a relatively busier unit in the hospital. (P3)

Even three or four recipients are enough for the workday of a nurse. Our workload is very high. (P5)

… It is a busy clinic. We need to be very careful because follow‐ups are frequent, treatments are frequent, the drugs are risky, and the patients are at risk. (P1)

If only we had better physical settings, if only we could be more care focused. Care is [already] our job, if only we could have more nurses. It is important to increase the numbers in these fields. (P8)

### 3.2 Theme: Symptoms of Compassion Fatigue

The symptoms of compassion fatigue among the participants were examined under four categories within the scope of this main theme: feeling of being unable to do anything, thoughts of inadequacy, putting oneself in the shoes of the helpless, and price of trying to do more.

#### 3.2.1 Feeling of Being Unable to Do Anything

The participants stated that in cases of rejection, graft loss, or patient loss, they felt that there was nothing they could do despite having provided the necessary care.

In some challenging cases, sometimes, one does not have anything left to do. I feel bad [in such cases]. It is not inadequacy, but inability to do anything… (P1)

We make the effort, but [a favorable outcome] does not occur; we make the effort, but it does not occur. Ultimately, it [feels like it] would have never occurred anyway… This saddens me because we make a lot of effort. (P7)

The creatine levels of the patient sometimes become worse, the patient feels sad, and I inevitably feel sad, too… One does not know what to say sometimes… (P3)

One does not know what to say when the patient [their body] rejects the kidney or liver they receive. This is one of the challenging points [of my job]. (P4)

For instance, the child got a kidney transplanted, and rejection developed. It needed to be removed, and a nephrectomy was performed again. I cried then, I mean, I was very sad… (P6)

#### 3.2.2 Thoughts of Inadequacy

The participants reported that they had feelings of inadequacy when they thought they were unable to provide sufficient care to their patients.

The essence of nursing is care. To be honest, I get sad when I am unable to provide care, [because] this is very important to me. Because the essence of nursing is care, doing these [performing care practices] pleases me. (P9)

… One feels bad when they fall short. Caring for fewer patients would also relieve us physically. It would make us feel more useful. (P1)

As a nurse, providing care makes me very happy; however, I experience much difficulty because we work under intense conditions. …I even feel inadequate sometimes, and this saddens me. This makes me unhappy. (P7)

…until you experience a loss. Then [when I experience a loss], as I feel I am not enough, my exhaustion turns into burnout. (P8)

#### 3.2.3 Putting Oneself in the Shoes of the Helpless

According to their statements, the participants put themselves and their families in the shoes of the patient in the context of the negative experiences of patients.

For example, as a mother, I feel the things felt by the child patient's mother. This exhausts me. (P1)

Our pediatric patients sometimes struggle [with their health problems] at very early ages. One thinks about their own child in their position (appears sad). We are intensely affected in the psychological sense. (P3)

Looking at the patients there, the thought that I could experience the same thing or whether my child or family could go through the same processes comes too frequently. You put yourself in their place, too. …but this affects us mentally. (P5)

…we, indeed, feel like patients and put ourselves into their shoes. We also get very sad. …because you make so much effort, you take care of everything from their treatment to boosting their morale and motivation, after a point, they become one of yours [family/circle]. (P6)

… I become really sad when I put myself in their place, when they are hopeless. (P7)

#### 3.2.4 Price of Trying to Do More

Regarding the process experienced by organ transplant patients, the participants said they felt the need to show more interest, sacrifice, compassion, and work more.

Organ transplantation is a more exhausting thing that requires more sacrifice. At this point, we beat ourselves up for so much monitoring and trying to not miss anything. (P5)

While talking to patients about coping methods, we are [the ones who are] affected. This reflects on us, too, in the same way. We are more worn out by this situation. (P6)

Some participants listed the problems they experienced as insomnia, fatigue, and malaise, using the following words:

Insomnia… Occasionally, you finish your shift and even sacrifice your sleep thinking ‘how was this patient, did I do that, did I provide this service, did I miss that…’ In addition to insomnia, fatigue, and malaise, several other conditions triggering these problems also develop. [These include] pain in the joints caused by standing for hours… (P5)

For example, once, I could barely sleep because I was saddened and affected [by my experiences] for an entire week… (P8)

… I might experience sleeping problems. …because we have an intense pace. For example, you lose sleep because you keep thinking about what you might have missed. (P12)

Using the following words, the participants explained that they were physically and mentally affected as a consequence of doing more than they were supposed to do for their patients:

… it [making an extra effort for patients] affects us both mentally and physically. (P7)

It affects us physically because we make an extra effort. [This affects us] mentally, while communicating, in the bodily sense… (P10)

I, for example, feel too much physical fatigue for this reason. …because we have too many patients. There is too much we need to keep up with, and we always try to take an extra step. Thus, your body gets exhausted. (P9)

[We are affected] physically like [experiencing] joint pain, muscle pain, or headache, in addition to fatigue, indisposition, restlessness, and sadness… (P11)

### 3.3 Theme: Management of Compassion Fatigue

The management of compassion fatigue by the participants was examined under three categories within the scope of this main theme: improving team spirit, designing individualized care, and embracing organ transplant nursing.

#### 3.3.1 Improving Team Spirit

The vast majority of the participants revealed that sharing between team members, talking about different topics, and organizing activities and meetings as a team alleviated the burden of compassion fatigue.

I can cope [with compassion fatigue] better by sharing with my friends. I feel more relieved by sharing these issues with my colleagues. One needs to plan and organize rather than complain. By sharing, talking about it, I actually succeed in [the management of] compassion fatigue. (P2)

Harmony among team members is very important. I believe we have great harmony. We occasionally talk about our compassion fatigue with our teammates. We respond to each other. …or we talk about other things; we make small talk. This is good for us; it is a positive thing. (P3)

If everyone gathers to share their feelings and express themselves as organ transplant nurses, they could feel relieved. (P8)

#### 3.3.2 Designing Individualized Care

The participants argued that being able to provide care without clinging to certain patterns could allow for better management of the process and more positive outcomes for both patients and nurses.

This is a professional field that requires knowing the patient very well psychologically, in addition to knowing their pharmaceutical details like which immunosuppressives they use or how many drugs they take. (P5)

Taking care of a patient feels like sowing a sapling into the soil for me. You spend labor, give water, grow it, take care of it, and when the patient picks themselves up, they get better, and everything is fixed… It is actually like a fruit tree. You eat its fruit, I mean, I am very relieved. I am happy in this sense. This actually gives me peace and happiness. (P9)

The participants noted that live donors are healthy individuals and emphasized the importance of protecting them from all forms of risk, educating them, and supporting them.

Well, our live donors are normally healthy individuals who donate their kidneys or livers. We observe the recipients in terms of rejection, while we also must monitor the donors closely in the postoperative period. (P3)

You collect an organ from a healthy individual, whom you must protect from all kinds of risks. Donor safety is the number one priority. (P8)

I believe we should not forget that they [live donors] are also people, they also need care, and they should also be supported. Being patients themselves, they also need information. (P12)

#### 3.3.3 Embracing Organ Transplant Nursing

The participants shared the following statements to express that they embraced their profession as organ transplant nurses and were aware that it is a special division of nursing:

Providing care to patients with organ transplants is a separate issue. …and this is acknowledged by all nurses. Everyone shows extra care and effort and works very professionally. I am fortunate to be working in such a place, and I am happy. Organ transplant nurses are truly special. They are more meticulous, special, and professional in all respects. (P9)

I believe organ transplant nursing is one of the distinguished fields of nursing. The transplant process, the education processes of donors and recipients, the challenges they can encounter in this process, potential problems… One needs to master the entire process. (P10)

Some participants described the satisfaction, happiness, and motivation caused by witnessing the transformation of patients with organ failure into healthy individuals again through organ transplantation as follows:

…yet [considering] the chronic disease they have experienced for years, [followed by] getting a new organ at the end… That individual will have an entirely different sense of satisfaction. (P5)

We have a heavy workload, but seeing the progress of the recovery of patients, their process of completely returning to a normal life… It makes me happy to see the result of my labor. (P5)

The recovery of patients in the post‐transplant process and the continuation of their lives makes me happy. I am satisfied doing this job and pleased with being a nurse. (P8)

I feel relieved when they get well and go out the door. This also makes me feel good in conscience. (P2)

Indeed, I feel motivated thinking of positive patients [patients with positive outcomes], those who are discharged after recovery, those who smile, those who leave smiling, those with a smile on their faces when they visit us… Those are the best for [alleviating] my compassion fatigue. (P3)

## 4 Discussion

In the literature, nurses are reported to be the health‐related profession with the highest predisposition to having compassion fatigue (Yee 2023). Very little is known about how compassion fatigue affects a nurse as a person, and indicators of how this affects the profession as a whole are also lacking (Gustafsson and Hemberg 2022).

The results of this study fill a gap in the literature on the effects of compassion fatigue on organ transplant nurses. The participants stated that despite feeling exhausted or burnt out due to their job and sometimes failing in their efforts, they provided their patients with the necessary care. Continued and regular emotional labor may lead to burnout or compassion fatigue, and this may reduce the quality of life and job‐related motivation of nurses (Kwak et al. 2020). Nurses experiencing high levels of compassion fatigue were found to have higher rates of making mistakes, providing low‐quality care, and intentions to leave their jobs (Jo et al. 2023). It is known that compassion fatigue affects nursing care and patient safety outcomes (Alharbi et al. 2020).

The categories of the theme “Causes of Compassion Fatigue” included the “heavy burden brought about by the responsibility of the transplanted organ”. In this context, it was found that the participants witnessed situations such as sorrow, fear, and loss. It was reported that providing care to terminal patients and individuals with special needs increased the compassion fatigue levels of nurses (İlter et al. 2024).

The organ transplant nurses who participated in this study described compassion as an innate human trait. They stated that being compassionate was a factor that gave rise to the emergence of compassion fatigue. Similarly, in the literature, psychiatric nurses defined compassion as an innate human trait (Bond et al. 2022). It was reported that nurses considered compassion to be a positive phenomenon, an empathic gift, and a quality a person is born with, rather than a learned method (Gustafsson and Hemberg 2022). In this study, the participants reported that they provided long‐term care to patients and their families, and they developed connections with their patients. It was seen in a previous study that seeing a patient a few times a week for years led many dialysis nurses to become emotionally attached to the care and individual situation of that patient (Pehlivan Saribudak and Aydın 2024).

The participants in this study reported that they used acceptance of their situation and suppression of stress as coping mechanisms. Another study conducted with nurses showed that there were shortcomings in the provision of resources and support to prevent compassion fatigue (Salmond et al. 2019). It was seen that despite difficulties, most nurses desired to be healthy role models for patients, families, and society (Melnyk 2020). The participants of this study reported experiencing negative situations such as stress‐related weight gain, worry, mental fatigue, and anxiety in the process of providing care to organ transplant patients. The participants of the study performed by Williams et al. (2022) offered recommendations including working less than 40 h per week in a clinical environment, working at consistent hours without a rotation system, expressing their emotions, allocating time for exercise, and participating in support groups. The professional fatigue seen in nurses was associated with constant mental health problems, absenteeism at work, and worsening job performance (Cho and Steege 2021). The participants of this study did not report a desire to change their current clinics or their jobs as a whole due to compassion fatigue.

The participants of this study described the organ transplant clinic as a busy work environment with a fast‐paced workload and thought that compassion fatigue would be inevitable in such an environment. It was reported in the literature that multiple difficulties contribute to an increase in the risk of compassion fatigue in emergency service nurses (Pérez‐García et al. 2021). In this study, the participants defined work overload as an obstacle to the provision of care. Wang et al. (2023) listed some factors associated with compassion fatigue and job satisfaction among obstetrics and gynecology nurses as the work environment, work experience, and night shifts. In another study in the literature, increased numbers of patients receiving nursing care per day were associated with high compassion fatigue levels (Sahin et al. 2023). It was found that various interventions, including the ability to receive clinical support, information, support groups, and resting breaks, had varying degrees of effectiveness in reducing compassion fatigue in nurses (Berger et al. 2022).

In this study, the participants revealed that in the physical and mental sense, they were negatively affected by compassion fatigue. In the literature, it was demonstrated that compassion fatigue left a mark on nurses both personally and professionally (Gustafsson and Hemberg 2022). Straughair et al. (2019) claimed that the personal characteristics of nurses could support meaningful interactions between nurses and patients and help nurses maintain compassionate care in clinical settings. It was reported that compassion fatigue caused individuals to doubt their own values and have thoughts about leaving their profession (Peters 2018).

In this study, the participants reported that they could experience situations such as feeling bad, self‐questioning, and feelings of inadequacy in cases like loss or rejection in transplant patients. Nurses who experienced compassion fatigue were reported to have decreased willingness to provide care to their patients (Karabuga Yakar et al. 2023). Wang et al. (2022) demonstrated that intensive care nurses experienced acute stress disorder following frequent patient losses. Mottaghi et al. (2020) reported that 77% of nurses experienced compassion fatigue due to the feeling of guilt. El‐Ashry et al. (2023) stated that intensive care nurses who witnessed the pain and death of patients during resuscitation attempts could feel guilty, weak, and anxious. In another study, nurses stated that they were inadequate in terms of providing care to a patient who was in pain, and they were unable to show a sincere approach (Gustafsson and Hemberg 2022).

It was found in this study that the work lives of the participants affected their attitudes toward life in general, and they frequently approached patients by putting themselves or their families in the shoes of patients. In agreement with the results of this study, another study in the literature showed that the work lives of almost two‐thirds of pediatric intensive care doctors and nurses influenced their attitudes toward life in general, and as a consequence of their jobs, they were frequently or highly frequently protective of their family members (Panagou et al. 2023).

The results of this study indicated that compassion fatigue affected the health of nurses negatively, and the participants reported experiencing physical and mental health problems related to compassion fatigue. Likewise, nurses who participated in the study conducted by Nolte et al. (2017) reported experiencing symptoms of fatigue and insomnia. In the literature, it was noted that insomnia not only negatively affected memory, learning capacity, performance, and reasoning but also was associated with weight gain, obesity, and musculoskeletal system problems (Williams et al. 2022). As stated by Ondrejková and Halamová (2022), in studies of compassion fatigue, additional somatic experiences including nausea, loss of appetite, pain, and aches or difficulty falling asleep have been reported in participants.

The participants of this study emphasized the negative effects of compassion fatigue on their social lives. In a qualitative study examining the causes and outcomes of compassion fatigue from the perspective of nurses, it was seen that the participants experienced difficulty performing their jobs, reflections in their family and private lives, anxiety, stress, and in some cases, intentions to leave their jobs (Pérez‐García et al. 2021). Cho et al. (2022) reported a reduction in perceived control among nurses with high levels of acute fatigue over the individualized care provided to their patients, particularly in the context of decision‐making in patient care. The participants of this study, on the other hand, pointed out that fatigue did not affect patient care negatively, and the feeling of sacrifice motivated them to show compassion toward others. These views of the participants were compatible with the humanizing approach in the definition of “compassionate care” by Straughair  (2019).

In this study, the participants described having feelings such as peace, happiness, relief, and trust as a result of providing care and proper communication with patients. According to Chen et al. (2022), participants who had better qualifications in the fields of education, counselling, communication, and coordination were more qualified in coping with the problems of patients and had a higher likelihood of receiving positive feedback for creating solutions to the problems experienced by their colleagues.

The findings of this study cannot be generalized to all transplant nurses. Nevertheless, the data provide important clues about the factors affecting compassion fatigue and demonstrate that rejection, in particular, has an emotional impact on nurses.

## 5 Conclusions

In this study, we examined organ transplant nurses' experiences of compassion fatigue and identified various factors that may contribute to compassion fatigue within the limited sample size available. The findings indicate that patient rejection, in particular, can psychologically affect nurses, and that the organ transplant process can lead to consequences such as stress, negative social life, anxiety, mental fatigue, and worry. While these results suggest that compassion fatigue among organ transplant nurses is an issue that deserves attention, they provide preliminary information rather than a basis for broad generalizations. The results of this study can help raise awareness regarding the development of compassion fatigue in organ transplant nurses and promote supporting precautions aimed at reducing the risk of compassion fatigue.

## 6 Strengths and Limitations

The first limitation of this study may be considered its single‐center design. This situation limits the generalizability of the findings. The findings should be considered exploratory. Studies with larger and more diverse samples will contribute to a more comprehensive understanding of the topic. Additionally, most of the participants were women, and this may be considered a limitation in terms of the gender distribution of the sample.

## 7 Implications for Clinical Practice

The results of this study can help raise awareness regarding the development of compassion fatigue in organ transplant nurses and promote supporting precautions aimed at reducing the risk of compassion fatigue.

It is important to identify the early symptoms of compassion fatigue. In addition to awareness, professional boundaries, self‐care practices, and individual and organizational education programs about the issue can help prevent compassion fatigue.

Organ transplant nurses should be supported in their development and implementation of personal plans to manage stress and maintain balance.

## Author Contributions

Conceptualization: M.E., N.A.; Methodology: M.E., N.A.; Data Collection: M.E.; Data Analysis: M.E., N.A.; Writing – Original Draft: M.E., N.A.; Writing – Review and Editing: M.E., N.A. All authors have read and approved the final manuscript.

## Funding

The authors have nothing to report.

## Ethics Statement

To conduct the study, permission was obtained from Akdeniz University Clinical Research Ethics Committee (Approval No: 2022/574) and written permission from the hospital where the study would be carried out was obtained. In terms of the protection of the identifying information of the participants, the principles of the Declaration of Helsinki (World Medical Association 2013) were followed throughout the study. To protect their anonymity and privacy, codenames (e.g., P1, P2, …) were used instead of the real names of the participants.

## Consent

All participants provided verbal and written consent.

## Conflicts of Interest

The authors declare no conflicts of interest.

## Data Availability

The data that support the findings of this study are available on request from the corresponding author. The data are not publicly available due to privacy or ethical restrictions.
